# Impacts of Smoking Ban Policies on Billiard Hall Sales in South Korea Using Objective Sales Information of a Credit Card Company: Quasi-Experimental Study

**DOI:** 10.2196/50466

**Published:** 2024-04-17

**Authors:** Jin-Won Noh, Jooyoung Cheon, Hohyun Seong, Young Dae Kwon, Ki-Bong Yoo

**Affiliations:** 1 Division of Health Administration College of Software and Digital Healthcare Convergence Yonsei University Wonju Republic of Korea; 2 Department of Nursing Science Sungshin Women's University Seoul Republic of Korea; 3 College of Nursing Keimyung University Daegu Republic of Korea; 4 Department of Humanities and Social Medicine College of Medicine and Catholic Institute for Public Health and Healthcare Management The Catholic University of Korea Seoul Republic of Korea

**Keywords:** smoking ban policy, indoor sports facility, South Korea

## Abstract

**Background:**

Smoking ban policies (SBPs) are potent health interventions and offer the potential to influence antismoking behavior. The Korean government completely prohibited smoking in indoor sports facilities, including billiard halls, since the government revised the National Health Promotion Act in December 2017.

**Objective:**

This study aimed to examine the impact of the SBP on the economic outcomes of indoor sports facilities, particularly billiard halls.

**Methods:**

This study used credit card sales data from the largest card company in South Korea. Data are from January 2017 to December 2018. Monthly sales data were examined across 23 administrative neighborhoods in Seoul, the capital city of South Korea. We conducted the interrupted time series model using the fixed effects model and the linear regression with panel-corrected standard errors (PCSE).

**Results:**

The sales and transactions of billiard halls were not significantly changed after the introduction of the SBP in the full PCSE models. The R2 of the full PCSE model was 0.967 for sales and 0.981 for transactions.

**Conclusions:**

The introduction of the SBP did not result in substantial economic gains or losses in the sales of billiard halls. In addition to existing price-based policies, the enhanced SBP in public-use facilities, such as billiard halls, can have a positive synergistic effect on reducing smoking prevalence and preventing secondhand smoke. Health policy makers can actively expand the application of SBPs and make an effort to enhance social awareness regarding the necessity and benefits of public SBPs for both smokers and the owners of hospitality facilities.

## Introduction

### Background

It is well-known that exposure to secondhand smoke causes illness and death. The World Health Organization has estimated that tobacco smoking kills 7 million people per year globally, of which 890,000 are due to secondhand smoke [1]. In the United States, the prevalence of secondhand smoke exposure among nonsmokers diminished between 1988 and 2014, from 87.5% to 25.2%. However, there was no change in exposure between 2011-2012 and 2013-1014 periods, and about 1 in 4 nonsmokers were still exposed to secondhand smoke during the 2013-2014 period [2].

Previous studies have reported that South Korea has a high prevalence of tobacco smoking [3,4], but the prevalence of smokers had markedly decreased by 2021 [5]. However, the prevalence is still high compared to other Organisation for Economic Co-operation and Development member countries [6], which means that people are considerably exposed to health threats and risks of secondhand smoke.

Between 2007 and 2018, the exposure rate to secondhand smoke among Korean adults decreased by 10.7%, and the exposure rate to secondhand smoke in indoor working areas decreased by 34.5% [6]. The reduction in the exposure rates to secondhand smoke in indoor working areas and public regions was especially prominent after 2012, most probably due to the continuous expansion of nonsmoking zones [7].

The smoking ban policy (SBP) is a potent health intervention, offering the potential to influence antismoking behavior. There has been an increase in the number of SBPs in countries globally, including Australia, England, and the United States, aligning with an increase in knowledge about the risk of secondhand smoke [8]. Indeed, the introduction of SBP leads to a decrease in exposure to secondhand smoke, improves indoor air quality, protects workers, reduces adult and youth smoking levels, decreases hospitalizations for acute myocardial infractions, and promotes respiratory health [9-11]. Notwithstanding the benefits of SBP, owners of hospitality facilities, including restaurants, bars, and billiard halls, have vigorously opposed the policy to curb smoking in these places, arguing that SBPs will result in economic hardship for them. This argument suggests that a complete ban on smoking in these places would discourage people from dining out, potentially negatively affecting sales. However, there is much evidence from the United States, Korea, Australia, and European countries indicating that economic performance was not affected by SBPs [12-15].

In accordance with the global trends of implementing SBP, indoor sports facilities (eg, billiard halls) in South Korea were regulated by the SBP as completely nonsmoking areas since the Korean government revised the National Health Promotion Act to prohibit smoking in all indoor spaces in December 2017 [16]. This change in SBP has led owners of indoor sports facilities to feel that the policy may negatively influence their economic profit, despite no significant change in sales. The effectiveness of SBPs has been evaluated by studies in other countries, demonstrating whether SBP affects economic profit. Previous works have consistently highlighted the impacts of SBPs on sales in various indoor places, such as restaurants and bars. For example, a previous study that included a sample of all 88 counties in the state of Ohio demonstrated that there was no significant difference in bar and restaurant sales following a statewide SBP between border regions in Ohio and nonborder areas [17]. The SBP in Ohio did not differentially influence the sales revenue for bars and restaurants located in counties where the border is shared with 5 other non–smoke-free states, compared to those in nonborder counties. Another study [18] supported the evidence that the SBP did not significantly affect facility sales, as the overall impact on sales in bars was negligible. The SBP was related to an increase in sales in medium to large bars in the rural region of Ireland and a small reduction in sales among large bars in the urban areas. These findings from previous works support the evidence for justification of continued use of SBPs to prevent the general public from exposure to secondhand smoke. However, there is a lack of evidence of the effects of SBP on business revenues in indoor sports facilities, even though many studies have been steadily involved in such research based on other indoor places. Moreover, it is crucial to assess the effect of the SBP, determining whether it resulted in a positive or negative economic impact. The results of the economic impact are important to provide evidence to visitors and owners of indoor sports facilities.

### Objective and Hypotheses

To date, there has been little study on changes in business revenues of indoor sports facilities, especially billiards halls since the introduction of the SBP in South Korea. This study aimed to examine the impact of the SBP on the economic outcomes of indoor sports facilities, specifically billiard halls, using actual revenue data from the largest card company in South Korea. Based on the evidence that hospitality facilities’ sales were not affected by SBPs [14,19,20], we hypothesized that the introduction of the SBP does not significantly affect billiard halls’ sales.

## Methods

### Data

This study used sales data from the Shinhan Card Big Data Center. The data included Shinhan’s credit, debit, and check card sales information from January 2017 to December 2018. Shinhan Card holders were 12 million in 2015, representing 44.6% of the economically active population in South Korea [21,22]. Shinhan Card has the largest market share (21.7%) in South Korea as of 2017 [23]. In 2016, a total of 80% of all private consumption in South Korea was made through card payments. A payment method survey in South Korea reported that 94% of Seoul citizens had 1 or more credit cards and 98% of Seoul citizens had 1 or more debit or check cards in 2013 [24]. The cash transactions were not included in our data, but the correlation between sales information provided by Shinhan Card data and the retail sales information of Statistics Korea was 0.92 [25]. The correlation between the sales information from all card companies in South Korea and the data of Shinhan Card was 0.97 [25]. Our data are suitable for assessing the effect of the policy.

In total, 3 districts in Seoul were selected for this analysis; Nowon district (533,498 population in 2019; 35.44 km^2^), Secho district (430,697 population in 2019; 46.98 km^2^), and Songpa district (675,843 population in 2019; 33.88 km^2^). The population in Seoul was 9,729,107 in 2019, and the 3 districts selected for this study account for 16.9% of Seoul’s population and cover 19.2% of Seoul’s total area. Regional experts at the Seoul Institute selected the districts considering various factors, including the percentage of the population aged 20-59 years, health behaviors (eg, smoking, drinking, and obesity rates), income level, environmental factors (eg, park space per capita and the number of designated smoking areas), and the similarity of *z* score for each factor with the average values for Seoul.

The unit of analysis for this study was neighborhood-month. Neighborhood in South Korea is referred to as “dong,” which is a submunicipal-level administrative unit of a city. We aggregated the individual billiard hall data into the neighborhood level. As the unit of analysis does not involve human subjects, this study does not require approval from an institutional review board.

### Ethical Considerations

This research used aggregated sales data from billiard halls by region. It is not subject to ethical considerations.

### Variables

The dependent variables were sales per neighborhood-month and transactions per neighborhood-month. The total sales information was aggregated from credit, debit, and check card use. A total of 1100 Korean won (KRW) was exchanged for US $ 1.

**Figure 1:**
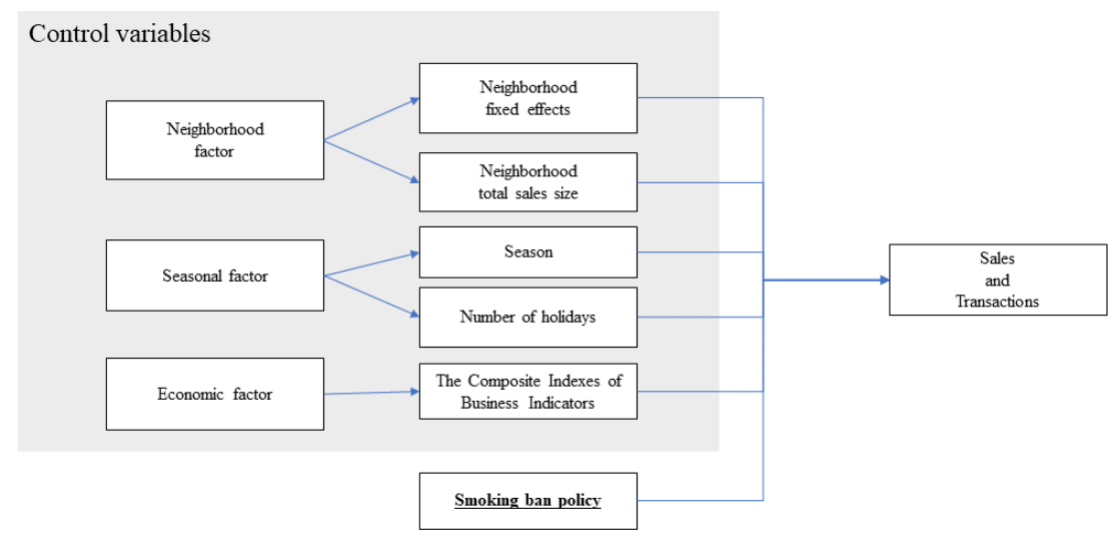
Research model and control variables of this study.

Based on previous studies on retail sales [14,26,27], the following factors were considered as the independent variables: socioeconomic factors of customers and region, seasonal factors, weather factors, employee factors, and overall economic status. The data consisted of neighborhood-month, making it impossible to consider the characteristics of individual customers and specifics of the store. Therefore, regional socioeconomic factors, seasonal factors, and economic factors were included as the independent variables in our study. The research model and control variables are shown in Figure 1.

Neighborhood total sales size represented the overall economic size of the neighborhood. It summarized all monthly credit, debit, and check card use in all business types except for online shopping, university tuition, insurance fees, taxes, and utility bills. As revenue is largely influenced by the total sales size of the neighborhood, it is an important covariate to analyze the billiard halls’ sales. Season and number of holidays per month were included as seasonal factors. Seasons were classified into spring, summer, fall, and winter because customers prefer to visit indoor facilities during summer and winter. Some billiard halls close on holidays, while others are crowded on holidays. The Composite Index of Business Indicators was included to adjust the overall economic condition in South Korea [28].

### Statistical Analysis

The Wilcoxon signed rank test was used to assess the mean difference between before and after introducing the policy. Our data were balanced panel data. Data constituted time-series–cross-section (TSCS) data, consisting of 24 months and 23 panels. Although the fixed effects (FE) model is commonly applied to analyze TSCS data, the assumptions of independence and identical distribution are prone to violation due to panel heteroskedasticity, contemporaneous correlation, serial correlation, and nonstationarity [29]. To identify these violations, we used various tests, including the Wooldridge test for serial correlation, the Pesaran cross-sectional dependence test for contemporaneous correlation, and the likelihood ratio test using Wiggins and Poi’s method [30] for panel heteroskedasticity. With the results of these tests, panel heteroskedasticity, serial correlation, and contemporaneous correlation were observed to be significant.

Therefore, a regression model with panel-corrected standard errors (PCSE) was the most suitable approach for our data [31]. The PCSE model is known to provide robust estimation for TSCS data when *T*≥15. Since the number of panels and the number of time points are almost the same, we conducted an FE model with robust standard errors to assess the robustness although there was a contemporaneous correlation.

The interrupted time series model, a quasi-experimental analysis, was used in the analysis [32]. Interrupted time series is a well-known method to analyze the effects of policies. It provides a policy effect by comparing the actual outcome with the potential outcome assuming that the baseline trend would be extended if the policy were not introduced [33].

The full regression model is as follows:



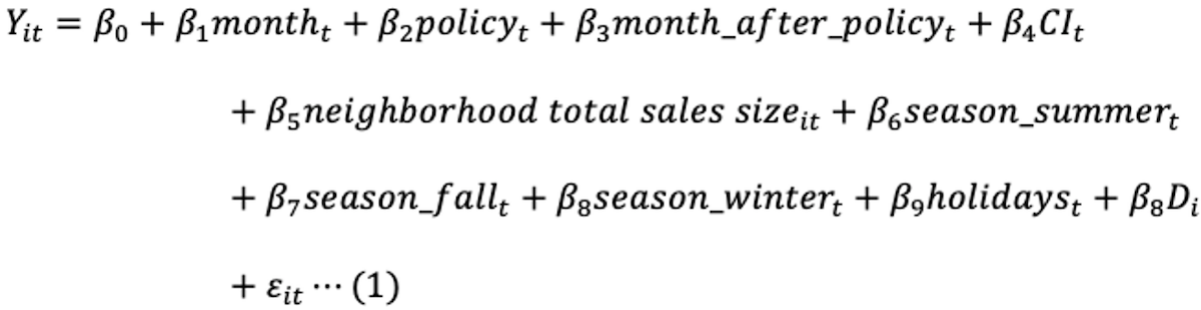



where *Y* is the dependent variable; *t* is the time period (month); *policy* is an indicator for the introduction of the policy introduced (0: before the introduction of the policy; 1: after the introduction of the policy); *D* is the dummy variables for neighborhood FEs; and *ε* is the error term. *β*_2_ and *β*_3_ represent the effects of the policy. *β*_2_ represents the level change due to the policy, and *β*_3_ represents the trend change after the policy was introduced, compared to the baseline time trend (*β*_1_). The effects of the policy can be calculated by considering both *β*_2_ and *β*_3_ after the time point when the policy was introduced. For example, the 1-year effect of SBP is calculated as follows: *β*_2_ + *β*_3_ × 12.

## Results

The general characteristics of the study data are shown in Table 1. The changes in sales information and neighborhood total sales size of billiard halls were insignificant in all 3 districts. Only the transactions of billiard halls in the Secho district significantly decreased, implied in the number of card payments. Since the sales in the Secho district did not change significantly, customers might be paying more per visit (Table 1).

**Figure 2:**
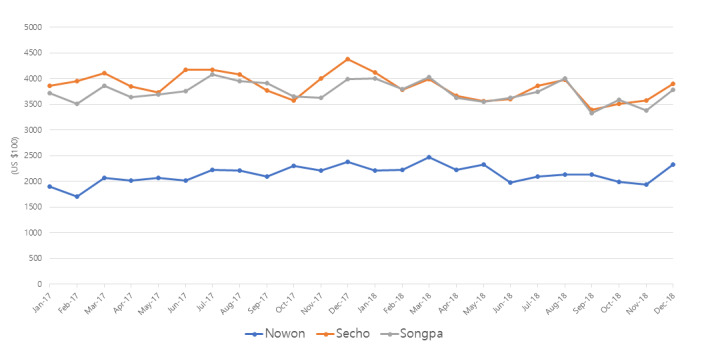
The trends of the monthly sales of billiard halls from January 2017 to December 2018.

Figure 2 shows the monthly sales trends of districts. The trends of billiard halls’ monthly sales in the 3 districts were almost flat (Figure 2).

**Table 1:** General characteristics of the study data by districts before and after the smoking ban policy.

Variables (in billiard halls)	Districts										
	Nowon (n=5^a^)				Secho (n=6^a^)				Songpa (n=12^a^)		
	Before	After	*P* value	Before		After	*P* value	Before		After	*P* value
Log (sales per neighborhood-month: US $100), mean (SD)	5.7 (1.1)	5.7 (1.2)	>.99	5.9 (1.3)		5.7 (1.7)	.06	5.4 (0.9)		5.4 (0.9)	.30
Log (transactions per neighborhood-month), mean (SD)	7.9 (1.2)	7.9 (1.2)	.31	7.9 (1.3)		7.7 (1.6)	.03	7.5 (0.8)		7.4 (0.9)	.11
Log (neighborhood total sales size: US $1 million), mean (SD)	7.3 (0.7)	7.2 (0.7)	.44	7.7 (1.3)		7.6 (1.7)	.69	7 (0.7)		7.1 (0.8)	.03

^a^The number of neighborhoods.

Table 2 shows the results of FE and PCSE regressions. PCSE regression with FE terms and control variables showed the highest *R*^2^ results in both the sales and transactions models. Our variables of interest were SBP and the month after the policy. In model 5, with the highest *R*^2^, the SBP’s coefficient (*β*_2_) was 0.0767. It shows that the dependent variable increased by 0.0767 constantly after the SBP implementation. The coefficient for the month after the policy (*β*_3_) was –0.0123. It showed that the dependent variables decreased by 0.0123 every month after the policy was introduced. However, neither variable was significant in all models for sales. The month after the policy, variables in transaction models were significant in models 1 and 2 (FE) as well as model 4 (PCSE), but they were not significant in model 5 (full PCSE). There was little evidence that the sales were affected by the SBP in billiard halls.

The Composite Index of Business Indicator was not significant for both sales and transactions, but it showed a positive relationship. This suggests that there may have been no significant macroeconomic issues from 2017 to 2018. The log of the neighborhood total sales size was significant for both sales and transactions; it showed that the billiard hall business was strongly affected by the economic status of its location. Compared to spring, only sales in winter were significantly higher. However, transactions were not significant in winter, compared to spring. It meant that people were likely to visit billiard halls and stay longer in the winter season. Transactions in summer were significantly higher than in spring (*P*=.049). The number of holidays did not significantly impact both sales and transactions.

**Table 2:** Effects of the smoking ban policy on the log of monthly sales and the log of monthly transactions of billiard halls (N=552).

Variable			Log (monthly sales)											Log (monthly transactions)								
			Model 1 (FE^a^)		Model 2 (FE)		Model 3 (PCSE^b^)		Model 4 (PCSE)		Model 5 (PCSE)		Model 1 (FE)			Model 2 (FE)		Model 3 (PCSE)		Model 4 (PCSE)		Model 5 (PCSE)
**Month**																						
	*β*_1_	0.0038		–0.0041		0.0054		0.0040		–0.0008		0.00697			–0.0002		0.0114		0.0083		0.0041	
	*P* value	.46		.53		.78		.58		.91		.15			.97		.59		.27		.51	
**Smoking ban policy**																						
	*β*_2_	0.0289		0.0977		–0.0034		0.0092		0.0767		–0.00611			0.0520		–0.0575		–0.0364		0.0237	
	*P* value	.54		.06		.97		.89		.22		.87			.13		.48		.57		.65	
**Month after the policy**																						
	*β*_3_	–0.0233		–0.0150		–0.0121		–0.0197		–0.0123		–0.0288			–0.0233		–0.0208		–0.0258		–0.0191	
	*P* value	.07		.08		.70		.07		.41		.02			<.01		.52		.02		.12	
**Composite Index of Business Indicator**																						
	*β*_4_	—^c^		0.0197		—		—		0.0561		—			–0.0035		—		—		0.0501	
	*P* value	—		.77		—		—		.66		—			.94		—		—		.63	
**Log (neighborhood total sales size)**																						
	*β*_*5*_	—		1.1220		—		—		1.0210		—			0.9971		—		—		0.9012	
	*P* value	—		<.01		—		—		<.01		—			<.001		—		—		<.01	
**Summer**																						
	*β*_*6*_	—		0.0452		—		—		0.0633		—			0.0450		—		—		0.0577	
	*P* value	—		.07		—		—		.07		—			.07		—		—		.05	
**Fall**																						
	*β*_*7*_	—		0.0308		—		—		0.0239		—			0.0024		—		—		–0.0040	
	*P* value	—		.30		—		—		.57		—			.93		—		—		.91	
**Winter**																						
	*β*_8_	—		0.0944		—		—		0.108		—			0.0488		—		—		0.0685	
	*P* value	—		.02		—		—		.04		—			.13		—		—		.11	
**Number of holidays**																						
	*β*_9_	—		–0.0045		—		—		–0.0055		—			–0.0017		—		—		–0.0028	
	*P* value	—		.32		—		—		.34		—			.61		—		—		.55	
Adjusting regional FE			Yes		Yes		No		Yes		Yes		Yes			Yes		No		Yes		Yes
*R* ^2^			0.059		0.581		0.679		0.936		0.967		0.118			0.650		0.791		0.959		0.981

^a^FE: fixed effects.

^b^PCSE: panel-corrected standard errors.

^c^Not applicable.

## Discussion

### Principal Findings

Despite the concerns of many people about the negative impacts of the SBPs on sales of indoor working areas [14,34], this study found that the sales and transactions in billiard halls were not affected by the SBP introduced in 2017 in South Korea. This finding supports previous research demonstrating that SBPs had no negative economic impact on sales of restaurants and bars in South Korea and other countries [14,34,35].

The first of the 3 reasons for no negative economic impacts on sales of billiard halls is that the social awareness of the need for public SBPs to prevent the harms of secondhand smoke has been increased due to mass media campaigns among both smokers and nonsmokers [36-39]. As smoking in public places becomes increasingly stigmatized, smokers may increasingly become aware that nonsmokers have the right to object to exposure to harmful passive smoking [36,40,41].

The second reason may be due to changes in the smoking population and increased preferences for no-smoking areas. The smoking prevalence among Korean adults aged ≥19 years decreased from 27.5% in 2010 to 20.6% in 2020 [42]. Smoking prevalence among men aged 30-50 years, who were the dominant population of smokers, decreased especially after 2015, when tobacco prices were raised from KRW 2500 (US $2.1) to KRW 4500 (US $3.8) and indoor smoking was banned in all businesses and restaurants [42-44]. A study found that Korean smokers in 2016 reported more positive perceptions of the effectiveness of expanded smoking bans and smoke-free policies compared to smokers in 2010 [40]. Therefore, the number of smokers who may complain regarding SBPs in billiard halls has decreased, and smokers who prefer smoke-free environments may still visit the billiard halls despite knowing that they are nonsmoking areas.

The third reason may be related to the indoor smoking room. According to a study conducted between 2018 and 2019, a total of 87% of billiard halls have indoor smoking rooms [41]. Based on the National Health Promotion Act in Korea, smoking rooms can be installed inside and outside of facilities, even if the facilities are smoking-free areas. Most smokers could use indoor smoking rooms despite the SBP in billiard halls, which may have resulted in no change in the sales of billiard halls. If there are no indoor smoking rooms or if rooms are far away from the playing area, smokers may decide not to smoke and focus on playing. The Ministry of Health and Welfare in South Korea reported that nonsmokers are more likely to be exposed to secondhand smoke in indoor public places with indoor smoking rooms and recommends closing indoor smoking rooms in all public facilities by 2025 [45]. Future research should examine the economic impact on indoor facilities and the consequences of secondhand smoke following the closure of indoor smoking rooms.

There are several additional benefits related to the SBP in billiard halls. First, SBPs reduce exposure to secondhand smoke, improve health outcomes, and reduce mortality due to smoking-related illnesses for both smokers and nonsmokers [36,46]. Second, smoking restrictions may lead to changes in smoking behavior among smokers, as they should spend additional time smoking due to SBPs, leading to an increase in quit attempts [46]. Third, the SBPs in billiard halls can lead to enhanced positive perceptions of the effectiveness of expanded smoking-free areas. A study found that past smokers and nonsmokers among owners, workers, and users in billiard halls and indoor golf clubs were more in favor of smoke-free areas after the SBP in 2017 compared to before the SBP was implemented [41].

Previous studies stressed price-based policy as the most effective means of reducing the consumption of tobacco [38,44], but the level of price increase in 2015 was insufficient to lead to a noticeable difference in South Korea [44,47]. Therefore, SBPs in public facilities, such as billiard halls, together with price-based policies, have positive synergistic effects in reducing smoking prevalence and preventing secondhand smoke [35,38,40,41]. This study has the strength of examining the impact of the SBP on billiard halls’ economic outcomes using actual revenue data from the largest card company in South Korea to provide a basis for enhancing the SBP.

However, there are some concerns in interpreting the findings of this study. First, this study could not adjust for the presence of indoor smoking rooms in billiard halls, which may be related to sales and transactions in these establishments. Future studies could compare sales between the billiard halls with and without indoor smoking rooms, or, in the case of closing indoor smoking rooms, compare sales before and after closure to provide a more robust evidence base for smoke-free policies. In addition, the inability to control for individual preferences and accessibility to indoor smoking rooms is one of the limitations of this study. Smokers might prefer to play pool rather than smoke, even though they are aware of the ban in billiard halls. Alternatively, smokers may not be aware of the existence of an indoor smoking area, or even if they are, they may choose not to use it while playing pool. Therefore, future studies should include individual preferences and adherence to smoking and smoke-free areas as well as environmental constraints, such as the presence and accessibility of indoor smoking rooms, in their analyses to determine if smoke-free policies have an impact on sales.

### Conclusions

This study examines the effects of the SBP in indoor sports facilities on billiard halls’ economic outcomes. Despite the worries of the owners of hospitality facilities, the SBP does not affect the sales of billiard halls. In addition to existing price-based policies, enhancing SBP in public use facilities, such as billiard halls, can have a positive synergistic effect on reducing smoking prevalence and preventing exposure to secondhand smoke. Based on this finding, health policy makers can actively expand the application of SBPs and make efforts to enhance social awareness of the need and benefits of public SBPs among both smokers and owners of hospitality facilities.

## Abbreviations

FE: fixed effects
KRW: Korean won
PCSE: panel-corrected standard errors
SBP: smoking ban policy
TSCS: time-series–cross-section

## Data Availability

The data sets generated during and analyzed during this study are available from the corresponding author on reasonable request.
